# Influence and Selection of Probiotics on Depressive Disorders in Occupational Health: Scoping Review

**DOI:** 10.3390/nu15163551

**Published:** 2023-08-11

**Authors:** José Antonio Picó-Monllor, Elena Sala-Segura, Romina Alin Tobares, Avelina Moreno-Ochando, Adrián Hernández-Teruel, Vicente Navarro-Lopez

**Affiliations:** 1 Department of Pharmacology, Pediatrics and Organic Chemistry, Faculty of Pharmacy, Miguel Hernández University (UMH), 03202 Elche, Spain; 2 Department R&D MATCH Biosystems, S.L. Edificio Quórum IV, Miguel Hernandez University Science Park, Avd. de la Universidad s/n, 03202 Elche, Spain; ali.tobares@matchbios.com (R.A.T.); avelina.mor@macthbios.com (A.M.-O.); adrian@matchbios.com (A.H.-T.); 3 MiBioPath Research Group, Faculty of Medicine, Catholic University of Murcia (UCAM), Campus de los Jerónimos n 135, 30107 Murcia, Spain; vnavarro@ucam.edu; 4 Clinical Microbiology and Infectious Disease Unit, Hospital Universitario Vinalopó, Carrer Tonico Sansano Mora 14, 03293 Elche, Spain

**Keywords:** occupational health, probiotics, depressive disorder, depression

## Abstract

Depressive disorders have a major impact on occupational health and are costly to the economy and the healthcare system. Probiotics are live, non-pathogenic micro-organisms that, when ingested in adequate amounts, can colonize the intestinal tract and confer health benefits on the patient. In recent years, numerous studies have described the potential usefulness of certain probiotic strains in the treatment and prevention of depressive disorders, with differing results. In order to evaluate the possible efficacy and safety of these microorganisms in preventing or ameliorating these disorders, we systematically searched the bibliographic databases MEDLINE (via Pubmed), EMBASE, the Cochrane library, Scopus and Web of science, using the descriptors “Occupational health”, “Probiotics”, “Depressive Disorder” and “Depression” and filters “Humans” and “Clinical Trials”. After applying our inclusion and exclusion criteria, 18 studies were accepted for review and critical analysis. Our analysis suggests that a combination of different probiotic strains, most of them from the genus *Bifidobacterium* sp. and *Lactobacillus* sp., could be a good mixture as an adjuvant in the treatment of depressive disorders for the working population.

## 1. Introduction

The World Health Organization (WHO) assembly proposes to develop national policies and action plans and to create institutional competencies for occupational health, ensuring, in collaboration with other relevant national health programmes, mental health [1]. According to the International Labour Organization (ILO), work helps to maintain good health as long as the worker’s physical and mental capacities are not overtaxed [2].

Research shows that health interventions in the workplace can reduce sickness-related absenteeism by 27% and the cost of healthcare for companies by 26% [1]. Globally, 12,000 million working days are estimated to be lost each year due to depression and anxiety, at a cost of USD 1 billion per year in productivity loss. Related health problems in this area cause a loss of GDP (gross domestic product) of between 4 and 6% in most countries. In 2019, an estimated 15% of working-age adults suffered from a mental disorder [1], and the ILO has established a number of occupational illnesses, including mental and behavioural disorders [3].

### 1.1. Depressive Disorders

According to the International Classification of Diseases (ICD) [4], depressive disorders are characterised by a persistent mood of sadness, irritability and lack of interest in daily activities, with a loss of pleasure accompanied by other cognitive, behavioural or neurovegetative symptoms that significantly affect the individual’s ability to function. Depression can be long-lasting or recurring and considerably impair a person’s ability to work, study or to cope with everyday life. In its most severe form, depression can lead to suicide [5,6,7]. Depressive disorders represent one of the leading causes of disability in the world, affecting 6.8% of the adult population, with a higher prevalence in women than in men [8]. During the COVID-19 pandemic, 23% of frontline healthcare workers suffered from depression and anxiety, and 39% suffered from insomnia. These professions are prone to depressive disorders, with an increased risk of suicide worldwide [9].

Psychological treatment is the first line against depressive disorders. This can be combined with antidepressants in cases of moderate and severe disorders. Effective psychological treatment may include behavioural activation, cognitive behavioural therapy, interpersonal psychotherapy and problem resolution treatment. While the most commonly used pharmacological treatments are serotonin reuptake inhibitors (SSRIs), serotonin-norepinephrine reuptake inhibitors (SNRIs) and tricyclic antidepressants [10,11,12,13], it should be borne in mind that all antidepressant drugs have a latency period of response, which can vary between two and four weeks, until the 5-HT1A receptor (serotonin receptor subtype located in presynaptic and postsynaptic regions) becomes desensitised. During this period, the patient, in addition to not noticing significant improvement, will perceive the possible side effects of the medication, which adds frustration, mistrust and possible non-adherence to the treatment [11,14]. Moreover, in most patients, the response to treatment is suboptimal, so that the dose has to be increased or combined with other antidepressants, further aggravating adverse symptomatology [15]. This insufficient response to antidepressants in a certain number of cases suggests that alternative or complementary medication, in addition to faster clinical response treatments, should be sought [16].

### 1.2. Microbiota

Microbiota are the set of microorganisms (bacteria, fungi, archaea, viruses and parasites) that reside in our body, which, in turn, can be differentiated into commensals, mutualists and pathogens [17,18]. Microbial communities can be found throughout the human body, the most complex and dense being the one inhabiting the digestive tract and, specifically, the large intestine (gut microbiota). It plays numerous roles in our organism, particularly the maturation and development of the central nervous system (CNS), as well as immune response development and modulation. It is referred to as the “second brain” of humans because of its regulatory effect on the central nervous system through neural, chemical and immune pathways [11,19,20]. Studies of the gut–brain axis (GBA) through the vagus nerve have provided essential insights into the healthy regulation of the hypothalamic–pituitary–adrenal axis (HPA), neuromodulation and neuronal plasticity [21]. This axis forms a network that includes the gastrointestinal tract, the enteric nervous system and the brain [20]. This gut microbiota play an essential role in the regulation of immune, endocrine and metabolic functions. Bacterial metabolites from this microbiota include the short chain fatty acids (SCFAs), made from fermentation of dietary fibre in the gut. Other enzymes and metabolites, such as tryptophan metabolites, gamma-aminobutyric acid (GABA), serotonin, dopamine, norepinephrine, acetylcholine and many neuropeptides, are produced by the microbiota present in the gut [16,19].

Given the ability of many probiotics to function as vehicles for the release of neuroactive compounds, such as classical neurotransmitters, they can be used as an adjunct therapy in the management of neurological disorders. For example, certain strains of Lactobacillus and Bifidobacterium secrete GABA, the main inhibitory neurotransmitter in the brain that regulates affective states, and increases levels of tryptophan, a precursor of serotonin, which suggests it is an antidepressant. The potential of some strains of Lactobacillus to produce acetylcholine, an essential neurotransmitter in several cognitive processes, such as learning and memory, means that they should be included as clear potential coadjuvant treatment, as psychobiotics [16].

Alterations in the gut microbiota, or dysbiosis, lead to a variety of diseases, such as inflammatory bowel disease, coeliac disease, metabolic syndrome, diabetes mellitus, colon cancer, as well as autism spectrum disorder, anxiety and neurodegenerative diseases [20,21]. This dysbiosis, due to increased permeability of the gut microbiota, induces decreased SCFA (short chain fatty acid) synthesis, HPA dysregulation and hypersensitivity of the vagus nerve, which predisposes one to depression [20,21] and, in some cases, to the progression or worsening of major depressive disorders (MDDs) [21].

### 1.3. Probiotics

Probiotics are live microorganisms that, when administered in adequate amounts, confer health benefits on the patient [22]. Recently, the International Scientific Association for Probiotics and Prebiotics [23] established a consensus document with a set of criteria for which microorganisms present in consumer products should be considered as probiotics:

- The microorganism in question must have been scientifically demonstrated to be a safe species that is supported by sufficient evidence of overall beneficial effect in humans.
- Evidence of its viability as a microorganism in human studies must be available.

Several studies [24,25] have proposed the concept of psychobiotics for probiotic bacteria, which, when consumed in adequate amounts, have a beneficial effect on mental health. The mechanisms of action by which bacteria exert their psychobiotic potential have not been fully elucidated. However, it has been found that these bacteria provide their benefits via the enteric nervous system or by stimulating the immune system. In addition, they affect psychophysiological markers of depression and anxiety. This can occur in three different ways: firstly, by affecting the stress response of the HPA axis and reducing systemic inflammation; secondly, through a direct effect on the immune system; and, thirdly, through the secretion of molecules, such as neurotransmitters, proteins and SCFAs [26,27].

Other studies link the intake of probiotics to the prevention and treatment of depression. The work of Bagga et al. [28] examined the clinical relevance of the gut–brain axis with the administration of a multi-species probiotic formulation for four weeks and associated it with changes in brain activation patterns in response to emotional memory tasks and emotional decision making, which were also accompanied by subtle changes in the gut microbiome profile. Akkasheh et al. [29] found that probiotic intake decreased depressive symptomatology as well as oxidative stress levels. In a study by Pirbaglou et al. 2016 [30], an improvement in immune function was observed in those taking probiotics, increasing the number of Natural Killer cells and lymphocytes. This said intake improved the bacterial composition of the gastrointestinal tract and, thus, behaviour and mental health. However, despite these previous works, a review by Vaghef-Mehrabany et al. [31] concluded that current studies are not sufficient to support or reject the antidepressant effects of probiotics.

The different studies on the subject and the differing results obtained only reinforce the belief that not all probiotics are valid, and that it is important to select probiotic strains that can influence the altered physiological processes of the illness targeted by the probiotic product in question [32]. What is important is that the selection of particular strains is based on objective criteria, preclinical studies or plausible hypotheses as to why a specific strain was selected instead of another. In this way, and with the support of clinical studies on the benefit of specific probiotics in specific pathologies, probiotic products can make a significant contribution to the health of the population and, thus, generate cost savings for health systems.

According to the WHO action plan, the loss of economic production due to mental disorders will amount to EUR 16.3 billion between 2011 and 2030. Depression alone accounts for 4.3% of the global burden of illnesses and is one of the leading causes of disability worldwide [33]. Therefore, new treatment options, such as probiotics, could help treat those affected by the illness and contribute to improving the mental health of the general population. Considering publications that demonstrate the benefits of certain probiotic strains in depressive disorders and their ability to stimulate the host’s immune response, we set out on this scoping review to identify and select probiotic strains that can prevent depressive disorders, decrease severity in those cases that eventually develop the illness and decrease the impact on occupational health.

## 2. Materials and Methods

### 2.1. Design

We conducted a descriptive study and critical analysis of papers retrieved through exploratory review according to the Preferred Reporting items for Systematic Reviews and Meta-Analyses extension for scoping reviews [34] (Supplementary Table S1).

### 2.2. Data Collection Sources

This review aims to carry out a critical and systematic study of works published on different databases by means of direct consultation and access via Internet to the following databases: MEDLINE (via PubMed), EMBASE, SCOPUS, Cochrane Library Plus and ISI-Web of Science (Institute for Scientific Information).

### 2.3. Information Processing

The document search was defined using the Thesaurus developed by the U.S. National Library of Medicine (Medical Subjects Headings—Mesh). Entry terms were also used. The terms “Occupational health”, “Probiotics”, “Depressive Disorder” and “Depression” were used as descriptors and free text in title and abstract. The final search equation was developed for use in the MEDLINE database, via PubMed, through the use of Boolean connectors and the filters “Humans” and “Clinical Trial”, with the following result: (“Depressive Disorder” (Mesh Terms) OR “Depression” (Title/Abstract) OR “Depressive Disorders” (Title/Abstract) OR “Depressive Symptoms” (Title/Abstract) OR “Emotional Depression” (Title/Abstract) OR (“occupational health” (Mesh Terms) OR “occupational health” (Title/Abstract) OR “Occupational Safety” (Title/Abstract) OR “industrial hygiene” (Title/Abstract) OR “employee health” (Title/Abstract))) AND (“Probiotics” (Mesh Terms) OR “Probiotics” (Title/Abstract) OR “Probiotics” (Title/Abstract) OR “Dietary Supplement” (Title/Abstract) OR “Synbiotic” (Title/Abstract) OR “Microbiota” (Title/Abstract) OR “Microbiotas” (Title/Abstract) OR “Dysbiosis” (Mesh Terms) OR “Dysbiosis” (Title/Abstract) OR “Dysbacteriosis” (Title/Abstract)).

The same strategy was adopted for the other databases mentioned above, taking into account their different characteristics. The search was carried out from the first available date until May 2023 (time of the last update). Additionally, as a secondary search and to reduce the number of papers not retrieved, the bibliographic lists for the articles selected in the main search were examined in order to identify previously undetected studies for the review.

### 2.4. Final Selection of Articles

The final selection of articles was made on the basis of the following inclusion criteria: papers had to be original clinical studies published in peer-reviewed journals, and there had to be a causal relationship between the intake of “probiotics” in “working-age subjects” and with “depressive disorders or “depression”, selecting those relevant with full text that could be retrieved, and which had to be written in English, Portuguese or Spanish. We excluded those that were not conducted on humans or that did not focus the intervention on probiotics in people of working age and that had no effect on depressive disorders or depression.

The selection of relevant articles was carried out independently by the authors of the present review (E.S.-S., J.A.P.-M., A.M.-O., A.R.T., A.H.-T., V.N.-L.). To validate the choice of articles for the review, it was established that the concordance assessment between these two authors (E.S.-S. and J.A.P.-M.) (Kappa index) needed to be greater than 0.80 (measure of very good concordance strength) [35]. Whenever this condition was met, possible discrepancies would be resolved through consultation with an expert in the field and subsequent consensus among the authors.

### 2.5. Methodological Quality Assessment

The quality of the selected articles was assessed on the basis of the CONSORT (Consolidated Standards of Reporting Trials) guidelines for reporting clinical trials [36], which contain a list of 25 essential items that should be described in the publication of these studies. For each selected article, one point was assigned for each item based on whether the article contained “1” or “0” with regards to the related information. If the evaluation of a particular item was not necessary, that point was not counted in the total (Not Applicable = NA). When an item was made up of several points, these were evaluated independently, giving the same value to each of them and then averaging (this being the final result for that item), so that in no case could the score of one point per item be exceeded.

### 2.6. Data Extraction

The control of the information extracted from the reviewed studies was carried out using double-entry tables that allowed for the detection of errors and correction by further consultation of the originals.

To determine how up to date the articles were, the Burton–Kebler half period (median age) and the Price Index (percentage of articles less than 5 years old) were calculated. The articles were grouped according to the variables under study in order to systematise and facilitate understanding of the results, coding the following data: first author of the bibliographical reference and year of publication, study design, country where the study was carried out, study population, period in which the work was carried out, what type of intervention took place and the results obtained.

## 3. Results

**Figure 1:**
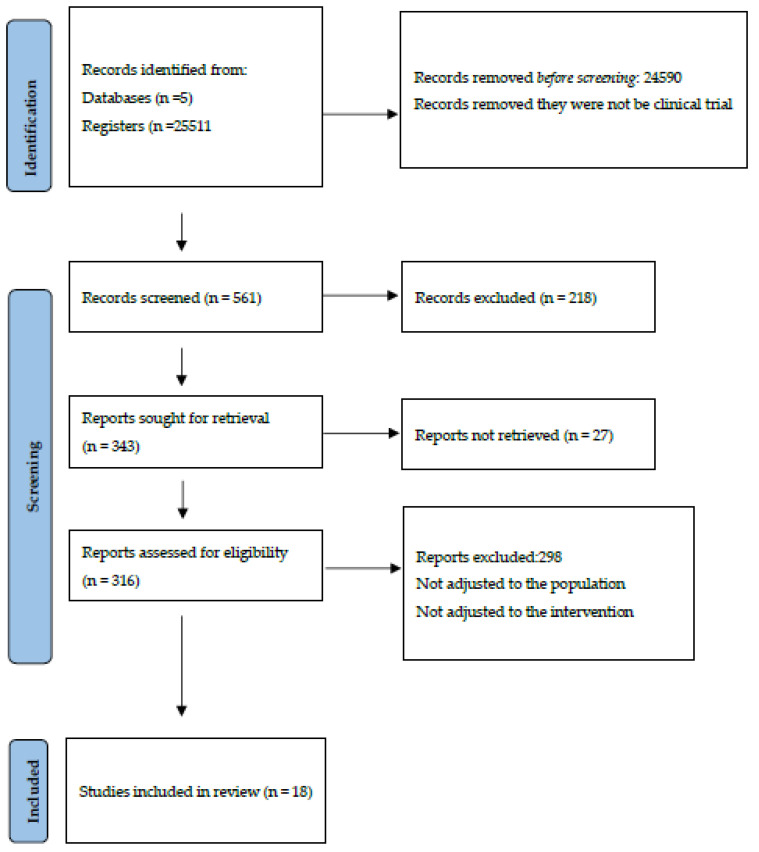
Identification and selection of studies according to Preferred Reporting Items for Systematic Reviews and Meta-Analyses (PRIMSA statement) [37].

Using the inclusion and exclusion criteria described previously, a total of 25,511 references were found: 23,318 (96.85%) on the Web of Science, 932 (3.87%) on Embase, 806 (3.35%) on Scopus, 448 (1.86%) on Medline (via Pubmed) and 7 (0.029%) on the Cochrane Library. After filtering duplicates, applying the inclusion and exclusion criteria and consulting the bibliographic listings of the selected articles (Figure 1), it was possible to select 18 papers for review and critical analysis (Table 1).

The agreement on the relevance of the selected studies among the authors, calculated using the Kappa index, was 85%. The selected articles presented, according to the Burton and Kebler semi-period or Burton–Kebler Index (BKI), a median of two years, with a Price Index (PI) of 88.88%. The papers were published between 2017 and 2023 [38,39,40,41,42,43,44,45,46,47,48,49,50,51,52,53,54,55]. The years 2020 [43,47,48,52] and 2021 [39,41,44,45], with four articles each, had the highest number of publications, followed by 2019 [40,46,53] and 2022 [49,51,55] with three publications, respectively. There were also two publications [42,54] for the year 2023 and only one publication per year for 2017 [50] and 2018 [38].

**Table 1:** Summary of reviewed studies on the relationship between probiotics and depressive disorders in healthy and ill subjects.

Author, Year	Design	Country	Patients	Population	Monitoring	Intervention Performed	Results
Bagga (2018) [38]	RCT	Austria	45 M/F 23/22 Age: 20 to 40 years	Healthy adult	4 weeks	3 g/day OmniBiotic^®^ Stress Repair (7.5 × 10^6^) cfu	Positive role of a multi-strain probiotic administration in modulating the behavior, which is reflected as changes in the FC in healthy volunteers
Bloemendaal (2021) [39]	Exploratory analyses	Netherlands	F: 56 Median age: 21.8 years	Healthy female	28 days	2 g/day Ecologic ^®^Barrier (2.5 × 10^9^) cfu	The increased relative abundance of the gut bacterial genus *Ruminococcaccae_UCG-003* correlated significantly with the positive effects of probiotics on stress-induced working memory changes
Chahwan (2019 [40]	Randomized triple blinded placebo controlled clinical trial	Australia	71 M/F: Age: years	Mild to severe level depression	8 weeks	4 g/day Ecologic^®^Barrier (2.5 × 10^9^) cfu	Participants in the probiotic group demonstrated a significantly greater reduction in cognitive reactivity compared with the placebo group, particularly in the mild/moderate subgroup
Chen (2021) [41]	Open label trial	Taiwan	11 M/F:3/8 Median age: 39.4 years	MDD	8 weeks	600 mg/day capsules *PS128* (3 × 10^10^) cfu plus AM	Depressive severity in patients with MDD significantly ameliorated, but markers of inflammation, gut permeability, and the composition of gut microbiota did not significantly change.
Freijy (2023) [42]	RCT	Australia	119 M/F: 11/108 Age: 18 to 65 years	Moderate psychological distress	8 weeks follow up (week 20)	2 capsules/day BioCeuticals © (12 × 10^9^) cfu plus high-prebiotic diet or 5 g/day High-prebiotic or synbiotic.	High-prebiotic diet benefits patients with moderate depressive disorders.
Heidarzadeh-Rad (2020) [43]	Post hoc RCT	Iran	78 M/F:23/55 Median age: 36 years	MDD	8 weeks	5 g/day CEREBIOME^®^ (10 × 10^9^)cfu and GOS (80%)	CEREBIOME^®^ exerted a beneficial effect in improving depression scores compared to placebo, while the effects of prebiotic supplement were not major.
Ho (2021) [44]	RCT	Taiwan	40 M/F 16/26 Age:20 to 40 years	Healthy adults with insomnia	30 days	850 mg/day capsules PS128 (3 × 10^10^) cfu	PS128 group showed significant decreases in BDI-II scores, fatigue levels, brainwave activity, and awakenings during the deep sleep stage.
Lee (2021) [45]	RCT	Seoul	122 M/F:39/83 Median age: 38.3 years	Healthy adults with subclinical symptoms of depression, anxiety and insomnia	8 weeks	1000 mg/day capsules NVP-1704 (2.5 × 10^9^) cfu	NVP-1704 group had a more significant reduction in depressive symptoms at four and eight weeks of treatment, and anxiety symptoms at four weeks compared to the placebo group
Nishida (2019) [46]	RCT	Japan	60 M/F: 41/19 Median age: 25.3 years	Healthy young adults	24 weeks	2 tablets/day Heat-inactivated *Lactobacillus gasseri CP2305* (1 × 10^10^) cfu	*Lactobacillus gasseri* CP2305 may be beneficial for young adults experiencing stressful conditions
Reininghaus (2020) [47]	RCT	Austria	61 M/F: 14/47 Median Age: 41.5 years	Depressive disorders	28 days	3 g/day Omnibiotic Stress Repair^®^ (7.5 × 10^9^) cfu and biotin vitamin B7 125 mg plus in addition to AM.	Probiotic plus B7 plus AM improved microbiota composition in depressive subjects
Reiter (2020) [48]	Monocentric RCT	Austria	61 M/F: 14/47 Median age: 41.5	MDD	4 weeks	3 g/day Omnibiotic Stress Repair^®^ drink (7.5 × 10^9^) cfu and 125 mg biotin vitamin B7 plus in addition to AM	The probiotic group showed decreasing IL-6 gene expression Probiotics could be a useful additional treatment in MDD, due to their anti-inflammatory effects
Rode (2022) [49]	RCT	Sweden	22 M/F: 6/16 Median age: 24.2 years	Healthy subjects	4 weeks intervention periods	3 g/day Puraflor^®^ (3 × 10^9^) cfu/day	The probiotic mixture had subtle effects on psychological health, such as rates of depression and sleep patterns, as well as on markers of gut-brain interactions, such as serum serotonin concentrations.
Romijn (2017) [50]	RCT	New Zealand	79 M/F: 17/23 Median age: 35.5 years	Subjects Low Mood	8 weeks	1.5 g/day Cerebiome^®^ (3 × 10^9^) cfu/day	Probiotic group, those with high levels of vitamin D at baseline, experienced significant improvement in several psychological outcomes.
Schaub (2022) [51]	RCT	Switzerland	47 M/F: 20/27 Median age: 38.6	Depression	4 weeks	Vivomixx^®^ (9 × 10^9^) cfu 4 times a day + AM	An add-on probiotic treatment improves depressive symptoms and maintains healthy enterotypes, species richness and increases specific health related bacterial taxa.
Siegel (2020) [52]	Pilot study RCT	USA	79 M/F = 21/58 Median age: 19.5 years	Healthy undergraduate students	7days	800 mg/day capsules *Bifidobacterium longum* (4 × 10^10^) cfu	No significant reductions in stress, depression, or anxiety were observed after 1 week
Smith Ryan (2019) [53]	RCT	USA	33 M/F:-/33 Median age: 30.5 years	Healthy female shift-workers	6 weeks	4 g/twice day Ecologic ^®^Barrier (2.5 × 10^9^) cfu plus 10g prebiotic W117	Reductions in anxiety and fatigue were greater in PRO than PLA
Yamanbaeva (2023) [54]	RCT	Switzerland	32 M/F: 14/18 Median age: 37	Depressive disorders (ICD-10)	4 weeks	Vivomixx^®^ (9 × 10^9^) cfu 4 times a day plus AM	Probiotic is suggested to prevent neuronal degeneration along the uncinate fasciculus and alter fronto-limbic rsFC, effects that are partly related to the improvement of depressive symptoms.
Schneider (2022) [55]	RCT	Switzerland	43 M/F: 17/26 Median age: 38.6 years	MDD	4 weeks	Vivomixx^®^ (9 × 10^9^) cfu 4 times a day plus AM	Additional probiotic supplementation enhances verbal episodic memory and affects neural mechanisms underlying impaired cognition in MDD

M/F: number males and females. ICD-11-10: International Classification of Diseases. CFU: Colony-forming units. MDD: Major depressive disorders OmniBiotic^®^ Stress Repair: *L. casei* W56, *Lactobacillus acidophilus* W22, *Lactobacillus paracasei* W20, *Bifidobacterium lactis* W51, *Lactobacillus salivarius* W24, *Lactococcus lactis* W19, *Bifidobacterium lactis* W52, *L. plantarum* W62, *Bifidobacterium bifidum* W23. Vivomixx^®^: *Streptococcus thermophilus* NCIMB 30438, *Bifidobacterium breve* NCIMB 30441, *Bifidobacterium longum* NCIMB 30435 (Re-classified as *B. lactis*), *Bifidobacterium infantis* NCIMB 30436 (Re-classified as *B. lactis*), *Lactobacillus acidophilus* NCIMB 30442, *Lactobacillus plantarum* NCIMB 30437 (re-classified *Lactiplantibacillus plantarum*), *Lactobacillus paracasei* NCIMB 30439, *Lactobacillus delbrueckii* subsp. *bulgaricus* NCIMB 30440 (Re-classified as *L. helveticus*). AM: Antidepressant or Antipsychotic medication. Ecologic^®^Barrier: *Bifidobacterium bifidum* W23, *Bifidobacterium lactis* W51, *Bifidobacterium lactis* W52, *Lactobacillus acidophilus* W37, *Lactobacillus brevis* W63, *Lactobacillus casei* W56, *Lactobacillus salivarius* W24, *Lactococcus lactis* W19 and *Lactococcus lactis* W58. Cerebiome^®^: *Lactobacillus helveticus* R0052 strain I-1722 and *Bifidobacterium longum* R0175 strain I-3470. GOS: Galactalogosaccharide. RCT: Double-blind randomized controlled trial. Bio Ceuticals^©^: *Bifidobacterium bifidum* (Bb-06), *Bifidobacterium animalis* subsp. *lactis* (HN019), *Bifidobacterium longum* (R0175), *Lactobacillus acidophilus* (La-14), *Lactobacillus helveticus* (R0052, *Lactobacillus casei* (Lc-11), *Lactobacillus plantarum* (Lp-115), *Lactobacillus rhamnosus* (HN001. FC: Functional connectivity. NVP-1704: mixture of *Lactobacillus reuteri* NK33 and *Bifidobacterium adolescentis* NK98. PRO: Ecological^®^ barrier plus resistant maize starch. PLA: Placebo. W117: Resistant maize starch. Puraflor^©^:, *Lactobacillus helveticus* R0052 *Lactiplantibacillus plantarum* R1012 and *Bifidobacterium longum* R0175. BDI: Beck Depression Inventory. PS 128: *Lactobacillus plantarum* (Re-classified *Lactiplantibacillus plantarum*).

Using the CONSORT questionnaire [36], scores ranged from a minimum of five (out of 25 items) to a maximum of 19, with a median of 14.8 (Table 2).

The majority of the studies were double-blind randomized controlled clinical trials (15.83%) [38,40,42,43,44,45,46,47,48,49,50,51,53]. The remainder were an open trial [41], an exploratory analysis [39] and a pilot study [52]. Out of the total, three studies were conducted in Austria [38,47,48] and Switzerland [51,54,55] each, with two in Australia [40,42], United States [52,53] and Taiwan [41,44], respectively. The remaining countries reviewed were featured in publications only once: South Korea [45], New Zealand [50], Netherlands [39], Iran [43], Japan [46] and Sweden [49]. All of the published papers reviewed were written in English.

The selected papers with the smallest sample sizes were those of Chen et al. [41] and Rode et al. [49], with 11 and 22 participants, respectively. In contrast, the papers by Freijy et al. [42] and Lee et al. [45], with 119 and 122 participants, respectively, presented the largest sample sizes.

The populations studied in the 18 selected studies consisted of people of both sexes. The age of the population included in the studies was between 18 and 65 years, with more or less severe depressive symptomatology [40,41,42,43,44,45,47,48,50,51,54,55], as well as healthy people [38,46,49,52]; of particular note, studies [39,53] focused exclusively on the female population. The follow-up period of the studies included in this scoping review ranged from a minimum of 7 days [52] to 24 weeks [46].

All the selected studies assessed the intake of different specific probiotic strains in the working population and their positive relationship with gut microbiota, biomarkers and clinical symptoms of depressive disorders (anxiety, insomnia, stress, depression, etc.). The species and strains used in the intervention were all Gram-positive, non-sporulating bacteria, and concentrations ranged from 10^6^ to 10^10^ colony-forming units (CFUs). The studies by Nishida et al. [46], Siegel et al. [52], Freijy et al. [42], Ho et al. [44], Lee et al. [45] and Chen et al. [41] used a capsule as the dosage form, with different amounts between 600 and 1000 mg. In contrast, the pharmaceutical sachet format varying between 1.5 and 5 g was the most commonly used [38,40,43,47,48,50,51,54,55].

Most of the trials used different bacterial formulations containing specific probiotic strains, most of them including the genera *Lactiplantibacillus*, *Ligilactobacillus* and *Limosilactobacillus* (third one formerly *Lactobacillus*) [56]. The genera *Bifidobacterium* were used in 14 of the selected studies [38,39,40,42,43,45,47,48,49,50,51,53,54,55]. Among these genera are included *Lactiplantibacillus plantarum* (*L. plantarum*), *Lacticaseibacillus casei* (*L. casei*), *Lacticaseibacillus rhamnosus* (*L. rhamnosus*), *Ligilactobacillus salivarius* (*L. salivarius*) and *Limosilactobacillus reuteri* (*L. reuteri*). The specific mixtures included in the selected articles are:

OmniBiotic^®^ Stress Repair: *L. casei* W56, *Lactobacillus acidophilus* W22, *Lactobacillus paracasei* W20, *Bifidobacterium lactis* W51, *Lactobacillus salivarius* W24, *Lactococcus lactis* W19, *Bifidobacterium lactis* W52, *L. plantarum* W62 and *Bifidobacterium bifidum* W23.

Vivomixx^®^: *Streptococcus thermophilus* NCIMB 30438, *Bifidobacterium breve* NCIMB 30441, *Bifidobacterium longum* NCIMB 30435 (re-classified as *B. lactis*), *Bifidobacterium infantis* NCIMB 30436 (re-classified as *B. lactis*), *Lactobacillus acidophilus* NCIMB 30442, *Lactobacillus plantarum* NCIMB 30437, *Lactobacillus paracasei* NCIMB 30439 and *Lactobacillus delbrueckii* subsp. *bulgaricus* NCIMB 30440 (re-classified as *L. helveticus*).

Ecologic^®^Barrier: *Bifidobacterium bifidum* W23, *Bifidobacterium lactis* W51, *Bifidobacterium lactis* W52, *Lactobacillus. acidophilus* W37, *Lactobacillus brevis* W63, *L. casei* W56, *L. salivarius* W24, *Lactococcus lactis* W19 and *Lactococcus lactis* W58.

Cerebiome^®^: *Lactobacillus helveticus* R0052 (CNCM strain I-1722) and *Bifidobacterium longum* R0175 (CNCM strain I-3470).

Puraflor^®^: *Lactobacillus helveticus* R0052 (CNCM-I-1722, *Lactiplantibacillus plantarum* R1012 (CNCM-I-3736) and *Bifidobacterium longum* R0175 (CNCM-I-3470).

Bioceuticals^®^: *Bifidobacterium bifidum* (Bb-06), *Bifidobacterium animalis* subsp. *lactis* (HN019), *Bifidobacterium longum* (R0175), *Lactobacillus acidophilus* (La-14), *Lactobacillus helveticus* (R0052), *L. casei* (Lc-11), *Lactobacillus plantarum* (Lp-115) and *L. rhamnosus* (HN001).

NVP-1704^®^: *Limosilactobacillus reuteri* NK33 and *Bifidobacterium adolescentis* NK98.

The remaining studies used a single strain, *Lactiplantibacillus plantarum* PS128 [41,44] and heat-inactivated *Lactobacillus gasseri* CP2305 [46], while the work of Siegel et al. [52] used a specific strain of *Bifidobacterium longum* without including further information on the strain. Some of the studies also combined probiotics with antidepressant medication [41,51,54,55] and vitamin B7 [47,48]. Others combined probiotics with prebiotics [42,43,53], and the rest used probiotic intervention alone.

The results of the probiotic intervention and its effect on depressive disorders in both healthy and ill subjects were varied. These results are summarized below.

### 3.1. Probiotic Intervention in Healthy Subjects

Bagga et al. [38] demonstrated, in their work, a close relationship between the effects of the intervention with nine probiotic strains and behaviour, reflected in improved functional connectivity (FC) and neuroimaging readings. However, they did not observe any difference in structural connectivity (SC). Bloemendaal et al. [39] observed an increase in the relative abundance of the genus *Ruminococcaccae*_UCG-003 in the gut microbiota, which correlated significantly with the positive effects of another nine probiotic strains on stress-induced working memory impairment in healthy women.

The work of Siegel et al. [52] and Rode et al. [49] presented similar negative results. In the former, the intervention with the *B. longum* species did not produce significant changes in improvements or reductions in stress, depression or anxiety in university students. In the latter, results with a mixture of three probiotic strains were subtle in terms of psychological health, depression rates and sleep patterns, as well as markers of gut–brain interactions, such as serum serotonin concentrations. In contrast, Nishida et al. [46] improved stress conditions in young adults with an inactivated probiotic CP2305 intervention, and Smith-Ryan et al. [53] observed a greater reduction in anxiety and fatigue with the intake of nine probiotic strains in female shift workers (nurses, emergency personnel, etc.), although without significant differences when compared with results in the control group.

### 3.2. Probiotics in Patients with Depressive Disorders

Reiter et al. [48] and Reininghaus et al. [47] reported positive results after ingestion of a mixture of nine probiotic strains together with vitamin B7 (biotin) in patients medicated with MDD. The probiotic mixture decreased interleukin 6 (IL-6) gene expression and improved the microbiota with an increase in potentially beneficial bacteria, such as *Ruminococcus gauvreauii* and *Coprococcus* 3.

The probiotic mixture of eight different strains together with antidepressant medication was used in the work of Yamanbaeva et al. [54], Schaub et al. [51] and Schneider et al. [55]. The results were prevention of neuronal degeneration along the uncinate fasciculus and alteration of the fronto-limbic rsFC, effects that are partly related to improvements in depressive symptoms, as measured by the Hamilton Rating Scale for Depression (HAM-D) and improvements in verbal episodic memory as well as modulation of neural mechanisms underlying impaired cognition with MDD. These clinical effects were accompanied by the maintenance of a healthy and diverse microbiota with an increase in specific health-related taxa.

With the same mixture of two different strains, the studies of Heidarzadeh-Rad et al. [43] and Romijn et al. [50] presented differing results. The former [43] showed a beneficial effect in improving depression scores compared to placebo. However, the latter [50] found no significant difference between the probiotic and placebo groups in any psychological improvement outcome.

The probiotic strain PS128 significantly improved the severity of depression in patients with MDD [41] and showed significant decreases in Beck’s Depression Inventory-II scores, fatigue levels, brain wave activity and awakenings during the deep sleep phase [44]. However, markers of inflammation, gut permeability and gut microbiota composition [41] and improved ANS functionality [44] did not change significantly.

Finally, the results of Chawan et al. [40] with the intervention of nine probiotic strains showed a significantly greater reduction in cognitive reactivity compared to the placebo group, especially in the mild to moderate subgroup, as did the results from the work of Lee et al. [45], where adults with subclinical symptoms of depression, anxiety and insomnia who consumed the NVP-1704 strain experienced a significant reduction in depressive symptoms after four and eight weeks of treatment, and in anxiety symptoms after four weeks, as well as a decrease in serum interleukin-6 (IL6) levels and an improvement in gut microbiota composition.

Other study by Freijy et al. [42], using a symbiotic combination of eight probiotic strains and prebiotics or only probiotics, showed no beneficial results on depressive symptoms. Only the prebiotic-rich diet was beneficial in patients with moderate depressive disorders.

## 4. Discussion

The study of the relevance/obsolescence of the chosen topic is quite pertinent and interesting. Out of the total amount of documents retrieved for this review, the majority were published in the last five years; the Burton–Kebler index (BKI) and the Price index (PI), respectively, presented values below the average and above the corresponding values in the area of health sciences. This data demonstrate the full relevance of the topic under study. In health sciences, an IBK of 9 to 12 years and a PI of 33.33% would be expected. [57].

Moreover, according to the degree of evidence and recommendation of the Scottish Intercollegiate Guidelines Network (SIGN) Grading Review Group [58], randomized controlled clinical trials are those that provide the most scientific evidence due to their consistent cause–effect relationship. The quality assessment of the studies included in this review using CONSORT was acceptable, with a mean of 14.8 out of 25. Therefore, the grade of recommendation was B (moderate evidence that the measure is effective and the benefits outweigh the harms).

Furthermore, English is the language of choice for the publication of most articles, which has become common in recent years since publishing in a different language is negative for the impact factor and citations of articles [59]. Moreover, the number of English-language journals available in the databases is currently very high [60].

The Comprehensive Mental Health Action Plan 2013–2030 presents, as main objectives, the implementation of promotion and prevention strategies, the strengthening of information systems, scientific evidence and research [33]. In this research study, we aimed to find out whether the use of probiotics could be an effective contributor to such a promotion and prevention strategy, as well as to improvements in depressive disorders. The 18 selected studies focused on various pathologies, such as anxiety, insomnia and depression. Most of these studies focus on the sick population and therapeutic intervention [40,41,42,43,44,45,47,48,50,51,54,55], while other studies, in a lesser amount, focus on prevention in healthy subjects [38,39,46,49,52,53] of working age.

The systematic review and meta-analysis by Huang et al. [61] confirmed that oral probiotic intake in workers under 60 years of age can effectively reduce scores for depression (Hamilton Rating Scale for Depression HAM-D). In addition, these scores were also improved in the different depressive stages of both healthy subjects and those with depressive symptoms. These data suggest, as concluded in the meta-analysis, that probiotics may be recommended for use in both non-depressed people and patients with depressive disorders. These results occur in the works of Bagga et al. [38], Nishida et al. [46], Smith-Ryan et al. [53] and Bloemendaal et al. [39]. However, the studies of Rode et al. [49], Romjin et al. [50], Siegel et al. [52] and Freijy et al. [42] did not show a significant reduction in improvements in depressive disorders.

The fact that, in the analyzed studies in this exploratory review, at least 28 different strains of bacteria were used successfully, either alone [41,44,46] or in different combinations [38,39,40,43,47,48,51,53,54,55], suggests that not only one specific strain or a single combination of probiotics will work but that many combinations may be a better treatment option. The use of different probiotic formulations, with no specific strain [52] or two [50] or three [49] probiotic strains alone or high doses of prebiotics [42] may account for the different results observed in these studies concerning improvements in depressive disorders. In selecting the appropriate combination of strains, important factors have to be taken into account, such as the fact that not all combinations are capable of obtaining positive results [32]. In this regard, the combination of *Lactobacillus helveticus* R0052 (CNCM-I-1722), *Lactiplantibacillus plantarum* R1012 (CNCM-I-3736) and *Bifidobacterium longum* R0175 (CNCM-I-3470) [49,50] had barely any effect on improving sleep patterns, depression or on serotonin concentration (biomarker of the gut–microbiota–brain axis). There were also no significant positive results in reducing stress, depression or anxiety with the *Bifidobacterium longum* intervention [52]. For the very young adult population included in this study, with an average age of 19.5 years, the use of a single species and the fact that the intervention lasted only seven days could be the reason for these weak results. In addition, the dosage used, lifestyle factors and the duration of the intervention could further influence the interpretation of the results [32].

Most of the studies included in this review used a combination of *Lactobacillus*, *Bifidobacterium* and/or *S. thermophilus* or *Lactococcus lactis* strains. The interventions with probiotic strains in healthy subjects [38,39,46,53] that showed positive results were *L. casei* W56, *L. paracasei* W20, *L. salivarius* W24, *L. plantarum* W62, *L. acidophilus* W37, *L. brevis* W63, *L. casei* W56, *L. salivarius* W24 *Lactococcus lactis* W19, *Lactococcus lactis* W58 and heat-inactivated *L. gasseri* CP2305 and *B. lactis* W51, *B. lactis* W52 and *B. bifidum* W23. Although the results of the selected studies are not homogeneous, it is once again corroborated that communication between the gut microbiota and the brain is a dynamic process, which can be modulated by a specific (probiotic) intervention, leading to changes in behaviour and brain function [38], and is associated with changes in frontal brain regions during cognitive control and with a microbiota increase in beneficial bacteria such as *Ruminococcaccae*_UCG-003 [39]. These results are in line with those obtained in clinical studies by Steenbergen et al. [62] and Papalini et al. [63], where a positive correlation was observed between intervention with these probiotic strains and cognition in difficult situations induced by acute stress. These probiotic strains also showed improvements in anxiety and fatigue reduction in female shift workers when combined with the prebiotic W117 [53].

Of particular note is the intervention with the heat-inactivated strain *L. gasseri* CP2305 and its benefits on young students under stress, with a 24-week treatment period [46]. Similar results were obtained in another study with the same inactivated strain, also including young chronically stressed students and with an intervention period of only 12 weeks [64].

On the one hand, intervention with the combination of 10 probiotic strains together with vitamin B7 in subjects with MDD [47,48] and in people with moderate to severe levels of depressive disorders [40], as well as the *L. reuteri* NK33 and *B. adolescentis* NK98 in subjects with subclinical symptoms of depression, anxiety and insomnia [45], improved the plasma levels of proinflammatory cytokines such as IL-6. The latter plays an important role in the pathogenesis of depression, and higher levels were associated with a poor prognosis and worse illness outcome [65,66], as well as with an improvement in negative thought patterns (cognitive reactivity) [40].

Other studies provide results of probiotic intervention on gut microbiota. In particular, an increase in potentially beneficial bacteria, such as *Ruminococcus gauvreauii* and *Coprococcus* 3 [47,48], and a decrease in proteobacteria [45] are reported.

Similarly, a combination of eight probiotic strains (*Streptococcus thermophilus* NCIMB 30438, *B. breve* NCIMB 30441, *B lactis* NCIMB 3043, *B.lactis* NCIMB 30436, *L. acidophilus* NCIMB 30442, *L. plantarum* NCIMB 30437, *L. paracasei* NCIMB 30439 and *L helveticus* NCIMB 30440), together with antidepressant medication, presented positive results correlating neuronal protection with improvements in depression symptoms (HAM-D), together with changes in bacterial diversity and an increase in healthy bacterial taxa of the gut microbiota. Such probiotics could be a useful additional treatment in MDD or in subjects with symptoms of depression, anxiety and insomnia, due to their anti-inflammatory effects [15,30], and this reinforces the potential microbiota-related treatment approach as one of the accessible therapies in disorders within the sphere of depression.

The studies evaluating the effect of the probiotic composition *L. helveticus* R0052 (CNCM strain I-1722) and *B. longum* R0175 (CNCM strain I-3470) presented contradictory results. While in the study by Heidarzadeh-Rad et al. [43], this combination produced beneficial effects on Beck’s Depression Inventory (BDI) scores, in the work of Romjin et al. [50], no positive results were obtained with respect to depressive symptomatology. Similarly, the addition of the probiotic strain *L. plantarum* R1012 to this probiotic composition [49] in healthy subjects also showed no positive results on depression scores, sleep patterns or gut–brain interactions. These results may suggest that these probiotic strains have a greater effect in patients with MDD than in patients with mild symptomatology.

*B. bifidum* (Bb-06), *B animalis* subsp. *lactis* (HN019), *B. longum* (R0175), *L. acidophilus* (La-14), *L. helveticus* (R0052), *L. casei* (Lc-11), *L. plantarum* (Lp-115) and *L. rhamnosus* (HN001) were used as another composition in a study by Freijy et al. [42], where the results showed improvements in mood, anxiety and sleep disorders in adults without a clinical history with the intervention of a prebiotic-rich diet, while the symbiotic mixture (prebiotic plus probiotic) or probiotic alone did not improve mild to moderate psychological disorders in patients with depression. These results should be viewed with caution, as this was not a double-blind study and, therefore, the participants and researchers were not masked in the allocation of the dietary intervention, which could have influenced how participants perceived or reported symptoms, an important bias to consider when assessing the results and information collected in the different forms of Total Mood Disturbances (TMDs), Beck Anxiety Inventory (BAI), etc.

The results of the present review are limited by the shortcomings inherent to each reviewed study [67]. For example, the probiotics selected in the reference papers, as well as the dosage and duration of treatment, are not the same, and other interferences, such as diet and medication, could also have affected the results. Another possible limitation is the small number of articles [44,53] found that specifically assess the impact on occupational health of probiotic intervention on people under stress or with depressive disorders. The depression assessment forms chosen by the different selected studies are not always the same. Moreover, the population studied was not homogeneous (different countries, different diet and different microbiota, shift workers, full-time and part-time workers, students, with mild to moderate and severe depressive disorders, with and without medication, etc.) and, in some cases, the monitoring of the population does not allow the results to be interpreted as strongly as expected.

As a conclusion of this review, and because occupational health is responsible, among other objectives, for the prevention of occupational illnesses among workers, such as anxiety, depression, insomnia, etc., caused by their working conditions [68], these inconclusive results should reinforce the interest of future research on probiotics, and the existing knowledge can serve as a starting point for the selection of strains with functional properties in improvements in depressive disorders. The combination of some probiotic strains that have shown a benefit in mental health could be considered as an adjuvant therapy in the management of mental disorders.

## 5. Conclusions

Despite the heterogeneity of the results obtained in the studies included in this review, probiotics may provide an effective adjuvant therapeutic tool to improve mental disorders in adults within the framework of public health. There were virtually no adverse effects in the populations analyzed.

For all the reasons presented throughout this study, we suggest that a combination that includes some probiotic strains, *Lacticaseibacillus casei* W56 *Lactobacillus acidophilus* W22, *Lactobacillus paracasei* W20, *Ligilactobacillus salivarius* W24, *Lactiplantibacillus plantarum* W62, *Lactobacillus acidophilus* NCIMB 30442, *Lactiplantibacillus plantarum* NCIMB 30437, *Lactobacillus paracasei* NCIMB 30439, *Lactobacillus helveticus* NCIMB 30440, *L. acidophilus* W37, *Lactobacillus brevis* W63, *Limosilactobacillus reuteri* NK33, *Lactiplantibacillus plantarum* PS128, *Lactococcus lactis* W19, *Lactococcus lactis* W58, *Bifidobacterium lactis* W51, *Bifidobacterium lactis* W52, *Bifidobacterium bifidum* W23, *Bifidobacterium breve* NCIMB 30441, *Bifidobacterium adolescentis* NK98, *Bifidobacterium lactis* NCIMB 30435 and *Bifidobacterium lactis* NCIMB 30436, should be tested on workers and people of working age with depressive symptomatology using large-scale clinical trials to assess their efficacy in improving their illness and to identify the best dosage for treatment.

## Figures and Tables

**Table 2:** Assessment of the methodological quality of the studies analyzed by means of the 25 items of the CONSORT 2010.

	1	2	3	4	5	6	7	8	9	10	11	12	13	14	15	16	17	18	19	20	21	22	23	24	25	T	%
Bagga [38]	0.5	1	0.5	0.5	1	0.5	0	0	0	0	0.5	0.5	0	0.5	0	0	0.5	0.5	NA	1	1	1	1	1	1	8	32
Bloemendaal [39]	0.5	1	1	0.5	1	0.5	0.5	1	1	1	0.5	0.5	1	0.5	0	0	0.5	NA	0	0	1	1	1	0.5	1	11.5	46
Chahwan [40]	1	1	1	1	1	0.5	0.5	1	0	1	0.5	1	1	0.5	1	1	0.5	NA	1	1	1	1	1	0	1	17	68
Chen [41]	0.5	1	0	0.5	0	0.5	0	0	0	0	0	0.5	0	NA	0	0	0.5	NA	NA	1	0.5	1	1	0	1	5	20
Freijy [42]	1	1	0.5	1	1	0.5	0.5	1	0.5	0	1	1	1	0.5	1	1	1	0	1	1	1	1	1	0	1	17	68
Heidarzadeh [43]	1	1	1	1	1	0.5	0.5	1	1	1	0.5	0.5	1	0.5	1	1	0.5	NA	1	1	1	1	1	0.5	1	17	68
Ho [44]	1	1	0.25	0.5	1	0.5	0	0	0	0	0.5	0.5	1	0.5	1	1	0.5	NA	1	1	1	1	1	1	1	13	52
Lee [45]	1	1	1	1	1	0.5	0.5	1	1	1	1	1	1	0.5	1	1	0.5	1	1	1	1	1	1	0	1	20	80
Nishida [46]	1	1	0.5	1	0	0.5	0.5	1	1	0	0.5	1	1	0.5	1	1	0.5	1	1	1	1	1	1	1	1	17	68
Reininghaus [47]	1	1	1	1	1	0	0	1	1	0	0.5	1	1	NA	1	1	0	NA	NA	1	1	1	1	1	1	17	68
Reiter [48]	1	1	1	1	1	0.5	0.5	1	1	0	0.5	1	1	NA	1	1	0.5	NA	NA	1	1	1	1	1	1	17	68
Rode [49]	1	1	0.5	1	0.5	1	0.5	0.5	0.5	1	1	1	1	0.5	1	1	0.5	1	1	1	1	1	1	1	1	18	72
Romijn [50]	1	1	1	1	0.5	0.5	0.5	1	1	1	1	1	1	0.5	1	1	1	0.5	1	1	1	1	1	1	1	20	80
Schaub [51]	1	1	1	1	0.5	0.5	0.5	1	0	1	1	0.5	1	0.5	1	1	1	1	NA	1	1	1	1	1	1	19	72
Siegel [52]	0.5	1	0.5	0.5	1	0.5	0	0.5	0	0	0.5	0.5	0.5	00	1	1	0.5	0	NA	1	0	0	0	1	0	7.5	30
Smith [53]	0.5	1	0.5	1	0.5	0.5	0	1	1	1	0	1	1	0.5	0.5	0.5	0.5	1	NA	1	1	1	1	1	1	14	56
Yamanbaeva [54]	1	1	1	1	0.5	0.5	0.5	0	0	1	0.5	1	1	0.5	1	0.5	0.5	0.5	NA	1	1	1	1	1	1	14	56
Schneider [55]	1	1	0.5	1	0.5	0.5	0	1	0.5	1	0.75	0.5	1	0.5	1	0	0.5	0	1	1	1	1	1	1	1	14	56

0 = does not meet the item or any part of it; 1 = meets the item in full; 0 to 1 = partially meets the item. NA: Not applicable. T: Total score.

## Data Availability

Not applicable.

## Supplementary Materials

The following supporting information can be downloaded at: https://www.mdpi.com/article/10.3390/nu15163551/s1, Table S1: PRISMA checklist for scoping review.
